# Effects of an entomopathogenic fungus on the reproductive potential of *Drosophila* males

**DOI:** 10.1002/ece3.11242

**Published:** 2024-04-08

**Authors:** Aijuan Liao, Fanny Cavigliasso, Loriane Savary, Tadeusz J. Kawecki

**Affiliations:** ^1^ Department of Ecology and Evolution University of Lausanne Lausanne Switzerland

**Keywords:** fungal infection, male fertility, mating ability, pathogen virulence, seminal fluid protein

## Abstract

While mortality is often the primary focus of pathogen virulence, non‐lethal consequences, particularly for male reproductive fitness, are less understood; however, they are essential for understanding how sexual selection contributes to promoting resistance. We investigated how the fungal pathogen *Metarhizium brunneum* affects mating ability, fertility, and seminal fluid protein (SFP) expression of male *Drosophila melanogaster* paired with highly receptive virgin females in non‐competitive settings. Depending on sex and dose, there was a 3–6‐day incubation period after infection, followed by an abrupt onset of mortality. Meanwhile, the immune response was strongly induced already 38 h after infection and continued to increase as infection progressed. Latency to mate somewhat increased during the incubation period compared to sham‐treated males, but even on Day 5 post infection >90% of infected males mated within 2 h. During the incubation period, *M. brunneum* infection reduced male reproductive potential (the number of offspring sired without mate limitation) by 11%, with no clear increase over time. Approaching the end of the incubation period, infected males had lower ability to convert number of mating opportunities into number of offspring. After repeated mating, infected males had lower SFP expression than sham controls, more so in males that mated with few mates 24 h earlier. Overall, despite strong activation of the immune response, males' mating ability and fertility remained surprisingly little affected by the fungal infection, even shortly before the onset of mortality. This suggests that the selection for resistance acts mainly through mortality, and the scope for fertility selection to enhance resistance in non‐competing settings is rather limited.

## Graphical abstract

This study investigates the effects of *Metarhizium brunneum*, a fungal pathogen, on the reproductive potential of male *Drosophila melanogaster*, covering both the pre‐copulatory and post‐copulatory aspects. We focused on scenarios where males paired with receptive virgin females in non‐competitive environments and measured male's fitness through the course of infection. Despite strong activation of the immune response, males' mating ability and fertility remained surprisingly little affected by the fungal infection, even shortly before the onset of mortality.

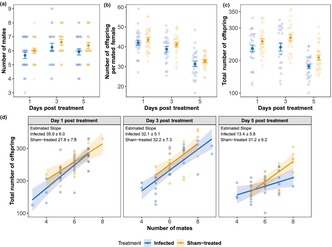

## 1 INTRODUCTION

Pathogens pose a ubiquitous challenge to individual health and population stability and persistence. The negative impact of a pathogen on host fitness (i.e., pathogen virulence), determines the strength of selection for resistance. In ecological and evolutionary contexts, pathogen virulence is most commonly quantified by the mortality rate of infected hosts. Although mortality puts an immediate halt on the host's reproduction, it is only part of the story when it comes to the pathogen's impact on host fitness. Even in the absence of mortality, or well before it occurs, pathogens can have a severe impact on host fitness by reducing its reproductive capacity. This can be a result of direct damage to reproductive tissues and other traits mediating reproduction (Polak, 1998; Sadd & Siva‐Jothy, 2006; Wilson & Denison, 1980), consumption of host resources by the pathogen, interference with the host's ability to acquire resources, or diversion of resources from reproduction to maintain somatic health (Gupta et al., 2022; Stahlschmidt et al., 2013). The impact of pathogens on the host's reproductive potential varies depending on pathogen types (Lower et al., 2023), host condition (Chambers et al., 2014; Lower et al., 2023) and the environment where the infection occurs (Bedhomme et al., 2004). Moreover, an infected host facing the prospect of impending mortality may increase its immediate reproductive effort, a phenomenon referred to as terminal investment (An & Waldman, 2016; Duffield et al., 2017; Zurowski et al., 2020). Thus, negative effects of pathogens on host reproduction are not universally expected; counterintuitively, pathogen exposure may even enhance reproduction in the short term.

Studies on pathogen virulence on host reproduction have primarily focused on females (Chadwick & Little, 2005; Hudson et al., 2020; Rose et al., 2022). Several studies have looked into the effects of infection on various aspects of male's reproductive biology (e.g., courtship (Kennedy et al., 1987; Pélabon et al., 2005), sexual ornaments (Dougherty et al., 2023; Longo et al., 2020), and sperm quality (Pham et al., 2022)). Yet, only limited number of studies investigated how infection affects males' mating success or overall reproductive success in the absence of mortality (De Lisle & Bolnick, 2021; Imroze & Prasad, 2011; Khan & Herberstein, 2022; Lehmann & Lehmann, 2000; Rittschof et al., 2013) and to what extent sexual selection contributes to the selection for resistance. Male's reproductive success is often primarily determined by access to females and their gametes (Bateman, 1948); thus, consequences of infection for male reproductive success will largely be mediated by responses of females to infected males. In other words, the effect of non‐lethal (or not‐yet‐lethal) infections on male reproductive success would to a large degree be mediated by sexual selection. Indeed, sexual selection is often postulated to favor males that are more resistant to pathogens (Adamo & Spiteri, 2005; Andersson & Simmons, 2006; Hamilton & Zuk, 1982), and this prediction rests on the assumption that infection impairs sexual competitiveness and attractiveness of males, at least of those that are more susceptible. Furthermore, the variance of reproductive success among males is often higher than among females (Janicke et al., 2016); hence, in contrast to selection mediated by mortality, selection for pathogen resistance mediated by reproduction can potentially be much stronger in males than females. However, the potential strength of this selection is limited by the degree to which the pathogen actually reduces male reproductive success prior to or without any mortality.

In this study, we used the fungal pathogen *Metarhizium brunneum* and *Drosophila melanogaster* as our experimental system to examine the impact of infection on traits contributing to male reproductive success. *Metarhizium* spores attach to *Drosophila* cuticle, penetrate it and reach the hemolymph, proliferate within the host and eventually kill the host when the life cycle of the fungus is completed, typically within 7–10 days (Lu et al., 2015; St. Leger & Wang, 2020). During proliferation, the fungus exploits the host for nutrients and energy and causes tissue damage through toxins and filamentous growth (Castrillo et al., 2005; St. Leger & Wang, 2020). Additionally, the activation of the immune system in response to the fungal infection (e.g., the production of antimicrobial peptides (AMPs)) disrupts cellular and organismal homeostasis (Tzou, De Gregorio, et al., 2002). Thus, both the fungal development within the host and the host's immune response imposes an increasing physiological burden on the host well before death. Mating is an expensive endeavor for *Drosophila* males, involving complex and energetically costly courtship and the production of seminal fluid proteins (SFPs). While courtship is important for convincing the female to mate, SFPs transferred from male to female during mating are important for securing the post‐copulatory sexual success and the outcome of fertilization (Avila et al., 2011; Wigby et al., 2020).

Here, we test how early and how strongly the burden of infection and immune response translates into a lower male reproductive success, and which of the multiple traits that contribute to pre‐ and post‐copulatory aspects of male reproductive success are affected. First, we conducted a survival assay to establish the timeline of pathogen‐induced mortality and measured the expression of AMPs following infection to examine the time course of the immune response. This also allowed us to test whether any effects of infection on male sexual performance coincide with the activation of the immune system, as would be expected if such effects were mediated by costs of the immune response. Then we evaluated how the progression of infection affects male sexual and reproductive potential in the absence of rival males and under high availability of potential mates (competitive success will be the subject of another study). To this end, we quantified number of mates, number of offspring per mated female, and the total number of offspring at different time points post infection but before the onset of infection‐induced mortality. While total number of offspring represents each male's overall reproductive success, number of mates indicates male's attractiveness or its ability to convince females to mate, and number of offspring per mated female demonstrates male's ability to fertilize eggs and to promote egg production and laying by the females. Lastly, we looked into the replenishment of five well‐characterized SFPs (*SP*, *Acp26Aa*, *Acp29AB*, *Acp62F*, and *Acp36DE*) after repeated mating. SFPs are transferred to females along with sperm during mating; their stock in accessory glands eventually becomes depleted after repeated mating (Hihara, 1981; Sirot et al., 2009), and they are costly to produce (both time‐ and energy‐wise). Along other >200 SFPs, they have important effects on post mating processes such as female receptivity (*SP*), ovulation (*SP*, *Acp26Aa*), oogenesis (*Acp62F*), sperm storage (*Acp29AB*), and sperm competition (*Acp29AB*, *Acp62F*, and *Acp36DE*) (Avila et al., 2011; Chapman, 2001). Quantifying SFP replenishment allowed us to investigate the impact of infection on male's non‐behavioral component of reproductive effort. We hypothesized that the progression of infection would negatively affect various components contributing to male's reproductive success but only when the infection is established within host and when the immune system has been fully activated.

## 2 MATERIALS AND METHODS

### 2.1 Fly stock

Flies used in the experiments originated from a laboratory‐adapted outbred population of *Drosophila melanogaster*, originally collected in Valais (Switzerland) in 2007. All flies were raised at a controlled density (~200 eggs on 40 mL food) and maintained at 25°C, 55% relative humidity and 12L:12D photoperiod on standard yeast–sugar–cornmeal–agar media with nipagin. When needed for experiments, virgin flies were collected 6–8 h post emergence and maintained in single‐sex groups until used in the experiment. Female virgin status was further confirmed by the absence of larvae in the food media. All fly transfers were done under light CO_2_ anesthesia.

### 2.2 Pathogen origin and infection protocol

The pathogen used in this experiment is *Metarhizium brunneum* KVL 03‐143 (Ma275, previously known as *M. anisopliae*, but now separated as a sister species (Bischoff et al., 2009); a generous gift from Nicolai Vitt Meyling, University of Copenhagen). The fungus was grown on Sabouraud dextrose agar (SDA) for 10 days at 26°C, after which spores were harvested and suspended in 0.05% Triton X‐100 (#9036‐19‐5; Sigma‐Aldrich). The concentration of spores was determined using a Neubauer hemocytometer. For the infection treatment, adult flies were dipped in groups of 10–15 for 30 s in 2 mL spore suspension of desired concentration. Males assigned to sham treatment were treated the same way but with spore‐free 0.05% Triton X‐100 (protocol adapted from (Ugelvig & Cremer, 2007)). Infection and sham treatment were done between 18:00–18:30 on the day before the experiment (i.e., Day 0). Measures on any day post treatment were done at 8:00 on the day of experiment, meaning that measures for Day 1 post treatment corresponds to around 14 h post treatment and subsequent measures were conducted every 24 hours.

### 2.3 Post‐infection mortality

To establish the timeline of infection‐induced mortality and to investigate whether it differs between the sexes, we conducted a post‐infection survival assay. Three spore concentrations in the infective suspension (10^6^, 10^7^, 10^8^ spores/mL) were used to understand how the dose affects fly mortality. Non‐virgin flies were subject to infection or sham treatment at the age of 3–4 days post emergence. They were then kept in groups of 10 in vials at 25°C and mortality was recorded daily until Day 16 post treatment. Any deaths of flies observed within the first 2 h were attributed to handling rather than infection; these individuals were therefore removed from the analysis (less than 1% of the treated flies). Mortality data were analyzed with a generalized linear mixed model (GLMM; binomial distribution, logit link) with the number of flies remaining alive (out of the initial number) as the response variable, day post treatment (DPT), dose (i.e., spore concentration), sex and their interactions as fixed factors and replicate vial identity as a random factor. DPT used in the model as a continuous variable was center‐scaled by subtracting the mean DPT value. This approach was chosen over typical survival analysis because a GLMM can better handle complex data structure and allows us to effectively find factors affecting mortality.

### 2.4 Activation of the immune system

The immune response mechanism in *Drosophila* has been extensively studied (Hanson & Lemaitre, 2020; Lemaitre & Hoffmann, 2007; Rai et al., 2023; Vlisidou & Wood, 2015). In *Drosophila*, one can easily track the immune response against *M. brunneum* by monitoring the expression of AMPs. To investigate the dynamics of the immune response post infection, we subjected 3–4 days old males to either *M. brunneum* infection (10^7^ spores/mL) or sham treatment (Immune Assay 1). Treated flies were then kept in groups of 16 and 14 vials per treatment were set up. We randomly selected two vials from each treatment pool and then collected four samples of eight flies on each day post treatment until Day 5 post treatment. These samples were used to measure the expression of *Drosomycin*, an AMP regulated by the Toll pathway and active against fungi and Gram‐positive bacteria. Considering that fungal infection might disturb the host homeostasis and facilitate the proliferation of other microbes within the host, we also quantified the expression of another AMP, *Diptericin A*, which is regulated by the IMD pathway and targets Gram‐negative bacteria.

We then carried out another immune response assay (Immune Assay 2) to investigate if the activation of the immune system is affected by the dosage of *M. brunneum* spores. We collected 3–4 samples of 2–3 flies from three concentration treatments (10^6^, 10^7^, 10^8^ spores/mL) on each day post infection until Day 5 post infection.

Total RNA of samples from both immune response assays was extracted with the Total RNA Purification Plus Kit, following the manufacturer's protocol (#48400; Norgen Biotek). 100 ng RNA was converted into cDNA using the PrimeScript RT™ reagent kit with gDNA Eraser (#RR047B; TaKaRa Bio). Each cDNA sample was diluted 10‐fold prior to the RT‐qPCR. RT‐qPCR was performed in 10 μL reaction volumes, containing 5 μL of SsoAdvanced Universal SYBRGreen Supermix (#1725272; BioRad, Switzerland), 0.3 μM of each forward primer and reverse primer, and 2 μL of cDNA templates. Cycling conditions consisted of 30 s initial activation of the polymerase at 95°C, followed by 40 cycles with 15 s denaturation at 95°C, 30 s annealing, and extension at 60°C. Following amplification, a melting curve analysis was performed ranging from 60°C to 95°C with 0.5°C increments for 1 s each. qPCR amplifications were performed in duplicate for each sample using the QuantStudio 6 Flex system equipped with a 384‐well block. We repeated the qPCR for samples with a Δ*C*_t_ SD between the two technical replicates more than 0.3. We performed qPCR for *Drosomycin* and *Diptericin A*, and three reference genes (*αTub84B*, *eEF1α2*, and *RpL32*). All primers used in the experiment are listed in Table S1. The expression of target genes relative to the reference genes was calculated using Pfaffl (2001) method but without a calibrator group.

To analyze the log_2_‐transformed relative expression of the immune genes, we used a linear mixed model (LMM) with treatment (infected vs. sham‐treated for immune assay 1 data and three doses for immune assay 2 data), day post treatment (DPT; a continuous variable), quadratic effect of day post treatment, and their interactions as fixed factors, and vial identity (accounting for possible vial effects) as the random factor. DPT used in the model was center‐scaled. Pairwise comparison was done using the *contrast()* function in *emmeans* package (v.1.7.1‐1) (Russell, 2021), and *p*‐values were adjusted with the Holm‐Bonferroni method.

### 2.5 Mating ability and latency

To study how developing infection affects male physiological and behavioral capability to mate, we performed a mating latency assay with receptive 3‐day‐old virgin females in a non‐competing setup. 2–3‐day‐old virgin males were infected with *M. brunneum* (10^7^ spores/mL) or sham‐treated as described above, and subsequently kept in groups of 10 until used in the mating trials. These mating trials were performed at five time points (Day 1–5 post treatment). On the day before mating, we randomly selected *N* = 50 infected males and 50 sham‐treated males (i.e., five vials each) for the mating trials (any male was only used once). Then one virgin male and one virgin female were put into the mating vial but kept separated by a paper separator (Hollis & Kawecki, 2014). The observation started on the next morning with removal of the separator at lights‐on time (8:00) and lasted for 2 h (flies are most active during this period). The time elapsed between the separator removal and the start of the first observed mating in the vial was noted as mating latency. No fly mortality was observed during the experiment. This experiment was done in two experimental blocks.

We compared the mating latency of infected and sham‐treated males on each day post treatment with a mixed effects Cox's proportional hazards regression model with package *coxme* (v.2.2‐16) (Therneau, 2020). The model included the treatment (infected vs. sham‐treated), day post treatment (DPT; a continuous variable), their interaction and experiment block (*N* = 2) as fixed factors. Day of experiment (a categorical variable, *N* = 10; accounting for any potential variability in experimental conditions across different days) was included as a random factor. DPT used in the model was center‐scaled. Males that did not mate within the 2 h observation period were included as right‐censored observations in the model. Estimated ratio of mating rate (“hazard ratio”) and the 95% confidence intervals were then acquired with the *emmeans()* function in *emmeans* package.

### 2.6 Reproductive potential

To investigate how *M. brunneum* infection affects the male's reproductive potential (i.e., the maximum number of viable offspring a male can sire within a given timeframe), we coupled each male (infected or sham‐treated) with 10 4–5‐day‐old virgin females and gave them 3.5 h to mate. This assay was done at three time points (Days 1, 3, and 5) post treatment. Each male was only used once. After the mating period, we transferred females into individual food vials and each female was given 48 h to lay eggs before being removed from the food vial. On Day 12 following the female removal (the usual emergence time for this population is ~10 days after the eggs are laid), we counted the number of vials with offspring, which we took as a measure the number of females successfully inseminated by each male (referred to as number of mates in the analysis). We also counted the number of offspring emerged from each vial, thus obtaining the total number of each male's offspring. Two experimental blocks were done consecutively within 2 weeks, with *N* = 23–27 males per treatment and time point.

As we only collected data at three time points post treatment in this experiment, DPT was included in the analysis as a categorical variable. Number of mates of each male was the outcome of 10 binary events (female mates with the male or not), so we analyzed it with a GLMM (binomial distribution, logit link) with treatment, DPT (a categorical variable), their interaction and experiment block as fixed factors and male identity as a random factor. Number of offspring per mated female was calculated by dividing the total number of offspring by number of mates. We then analyzed this measure using a LMM with treatment, DPT (a categorical variable), their interaction and experiment block as fixed factors and day of experiment as a random factor. Then, total number of offspring was analyzed with a LMM including treatment, DPT (a categorical variable), their interactions and experiment block as fixed factors and day of experiment (*N* = 6) as a random factor. To test how number of mates affects overall male reproductive success, we modified the LMM analyzing the total number of offspring to include the number of mates and interaction terms involving the number of mates along with other variables included in the previous model as fixed factors. The relationship between number of mates and total number of offspring in the two treatments was compared with the *lstrends()* function in the *emmeans* package, and *p*‐values were adjusted with the Holm–Bonferroni method.

### 2.7 Replenishment of seminal fluid proteins

To investigate whether the fungal infection affects the SFP replenishment rate after repeated mating, we compared the gene expression level of the SFPs in infected and sham‐treated males from the assay described in the *Reproductive Potential* subsection, that is, after they have mated with multiple females. At the end of the mating period, each male was transferred to a fresh food vial and kept for 24 h before being collected, snap‐frozen in liquid nitrogen, and transferred to −80°C until RNA extraction. As each sample only contained a single fly (small biomass), in this assay, the total RNA was extracted using the RNeasy Micro Kit (#74034; Qiagen GmbH) following the manufacturer's protocol. RNA sample was reverse transcribed into cDNA using the PrimeScript RT™ reagent kit with gDNA Eraser (#RR047B; TaKaRa Bio). Ideally, 100 ng RNA would have been used for the cDNA conversion but in some samples, this amount was not obtained, so the maximum amount of RNA was taken (range = [51 ng,100 ng], mean = 91.52 ng). Each cDNA sample was then diluted 10‐fold. RT‐qPCR was performed and relative expression calculated in the same way as described in *Activation of the Immune System*. Primers for the reference genes and SFPs used in the experiment are listed in Table S1.

Reflecting the small amount of material obtained from single males and the individual variation, the SFP expression estimates were quite variable, with several apparent outliers. To identify the outliers, we fitted a LMM to log_2_ expression levels of each SFP, with treatment, DPT, their interaction and experiment block as fixed effects, and day of experiment as a random effect. From this model we obtained externally Studentized residuals with the *rstudent()* function of the *stats* package (v.4.1.2) (R Core Team, 2020). Across the five SFPs, we removed six data points out of 755 (five of which from the same sample, i.e., same individual) with Studentized residuals of an absolute value greater than 3.7. Under Student's *t* distribution with the number of degrees of freedom of the model (df = 143) and sample size (*N* = 151), the likelihood of obtaining one or more values above this threshold is *p* < .05.

To test whether *M. brunneum* infection affects SFP replenishment and whether different SFPs respond differently, we analyzed the relative gene expression of all five SFPs jointly. We fitted a LMM to log_2_‐transformed relative gene expression, with the identity of the SFP, treatment (infected vs. sham‐treated), day post treatment (i.e., day of mating; a categorical variable), their interactions and experiment block as fixed effects; male identity was included as a random effect.

Males used in this assay had mated a variable number of times, and the number of matings should affect SFP depletion and thus likely the investment in SFP replenishment. This may not only add variation to the SFP gene expression data but could also cause systemic differences between infected and sham‐treated males without infection affecting the capacity to invest in SFP investment, if these two groups mated with a different number of females. We thus tested the relationship between a male's investment in SFP replenishment and the number of females it had mated with 24 h prior to being collected. To facilitate this analysis, we combined the expression of all five SFPs into a single index aiming to estimate the overall investment of a male into SFPs. To obtain this index, log_2_ expression values of each SFP were zero‐centered (by subtracting the mean) and scaled by dividing by the residual standard deviation from each SFP‐specific model (which included relative expression of each SFP as response variable, number of mates, DPT, treatment, their interactions, and experiment block as fixed factors and day of experiment as a random factor). The index was then calculated by averaging these scaled values across the SFPs. By using the residual standard deviation from the SFP‐specific models, we took into account the different characteristics of different SFPs while using a combined index. We fitted a LMM with the combined SFP expression index as the response variable, number of mates (centered on the mean), DPT, treatment, their interactions, and experiment block as fixed factors and day of experiment as a random factor. The relationship between number of mates and SFP replenishment of the two treatments was compared with the *lstrends()* function in the *emmeans* package, and *p*‐values were adjusted with the Holm–Bonferroni method.

### 2.8 Statistical analysis

All statistical analyses described above were done with R (v. 4.1.2) (R Core Team, 2020) and R studio as IDE. Visualization of the results was conducted with package *ggplot2* (v. 3.4.1) (Wickham, 2016). Statistics of the (generalized) linear mixed models were attained using the *mixed()* function within the *afex* package (v.1.0‐1) (Singman et al., 2021), and *p*‐values were calculated using the likelihood ratio test.

## 3 RESULTS

### 3.1 Post‐infection mortality

**FIGURE 1:**
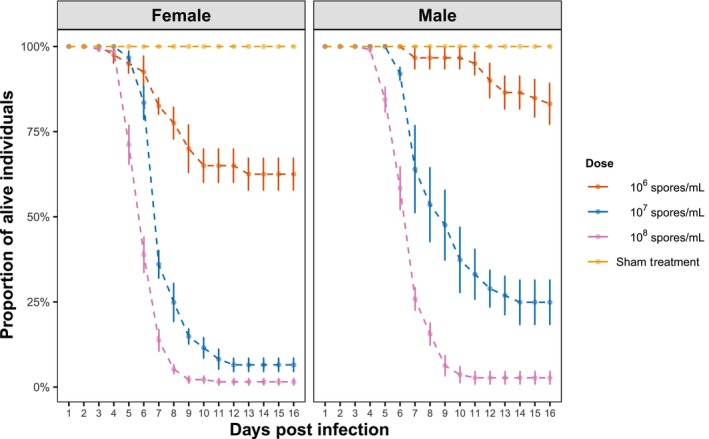
Post‐infection survival of flies following infection with different doses of *M. brunneum* (concentrations of spore suspension). Symbols are means ± SE.

For both females and males, there was a pathogen incubation period of about 3–6 days following the infection treatment (i.e., a period when fungal proliferation has not yet caused any mortality; Figure 1). Mortality was dose‐dependent, increasing with the concentration of *M. brunneum* spores (Figure 1; LRT, dose, $\chi_{2}^{2}$ = 99.8, *p* < .001; Table S2). At any given dosage, males had a lower mortality rate than females (Figure 1; sex, $\chi_{1}^{2}$ = 33.4, *p* < .001; Table S2), suggesting that males were less susceptible to *M. brunneum* infection than females.

### 3.2 Activation of the immune system

**FIGURE 2:**
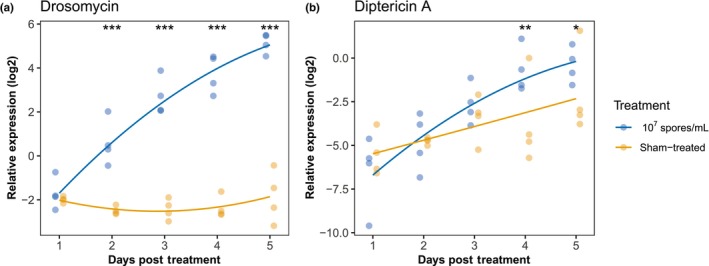
Relative expression of antimicrobial peptide genes, (a) *Drosomycin* and (b) *Diptericin A*, after *Metarhizium* infection (10^7^ spores/mL) or sham treatment. Data are from Immune Assay 1. Each dot represents a sample of eight males. Solid lines demonstrate the predicted values from the linear mixed models; significance level from pairwise comparisons are shown: ***p ≤ .001, **p ≤ .01, *p ≤ .05.

We examined the expression of *Drosomycin* (active against fungi and Gram‐positive bacteria) and *Diptericin A* (primarily induced by Gram‐negative bacteria) following the *M. brunneum* infection. Following treatment, the level of *Drosomycin* expression within infected males increased as the infection progressed (treatment, $\chi_{1}^{2}$ = 80.5, *p* = .002, treatment × day post treatment, $\chi_{1}^{2}$ = 59.2, *p* < .001; Table S3) and became significantly higher than that of the sham‐treated males starting from Day 2 post infection (Figure 2a). The expression of *Diptericin A* also increased over time (treatment, $\chi_{1}^{2}$ = 4.3, *p* = .039, treatment × day post treatment, $\chi_{1}^{2}$ = 7.8, *p* = .005; Table S3) and infected males had higher level of the *Diptericin A* expression starting from DPT 4 (Figure 2b). However, the maximum difference between infected and sham‐treated males was much lower for the expression of *Diptericin A* (about 2‐fold) and *Drosomycin* (about 100‐fold). Different dosages of *M. brunneum* spores activated the AMP expression to a similar magnitude (Figure S1; Table S4).

### 3.3 Mating ability and latency

**FIGURE 3:**
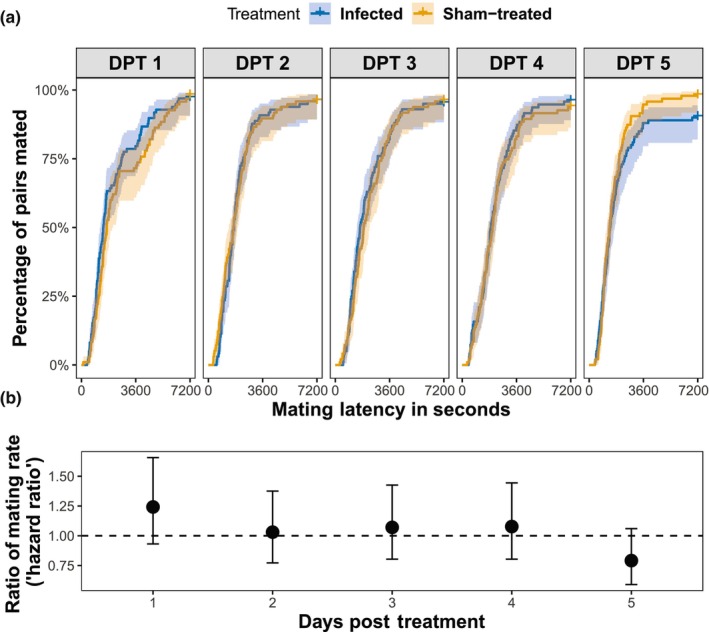
Effect of infection on mating latency of a male paired with a virgin female. (a) Cumulative proportion of pairs that initiated copulation in the course of 2 h observation period. Shadow indicates the 95% confidence interval. (b) Estimated ratios of mating rates of infected to sham‐treated on each day post treatment (hazard ratio from the mixed effects Cox's proportional hazards regression model and its 95% confidence interval).

No mortality due to infection was observed during the mating trials, which was consistent with the mortality of males infected with 10^7^ spores/mL not starting before Day 6 post infection (Figure 1). Nearly all infected males (≥90%) mated within the 2 h observation period; even on Day 5 post infection ~90% of the infected males mated, implying that infection has little effect on males' ability to mate during pathogen proliferation (Figure 3a; Figure S2).

Nonetheless, we detected a significant interaction between day post treatment (DPT) and treatment on the mating latency (Cox proportional hazard model with mixed effects, day post treatment × treatment, *p* = .034; Table S5). This can be seen as a decline in the ratio of mating rates (i.e., the “hazard ratio” from the Cox regression); even though the ratio was not statistically different from 1 on any day, it declined over time (Figure 3b). This implies that progression of the infection did have a slight negative effect on this aspect of male sexual performance.

### 3.4 Reproductive potential

**FIGURE 4:**
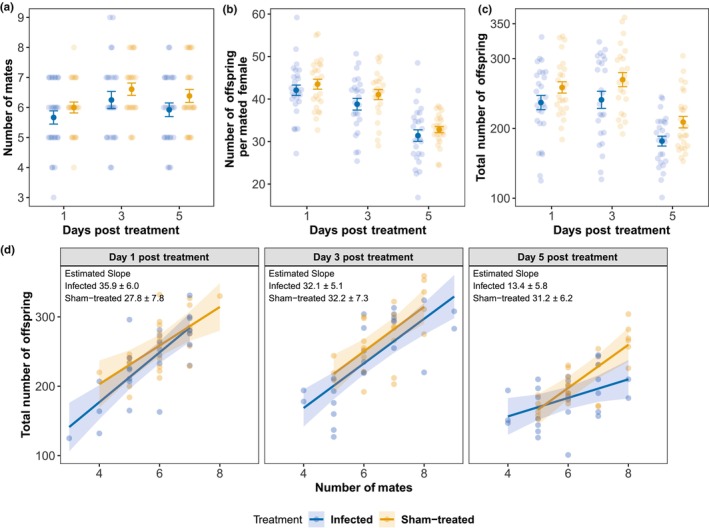
Reproductive potential of infected and sham‐treated males on Days 1, 3, and 5 post treatment. (a) Number of females successfully inseminated by each male (i.e., the number of mates); (b) Number of offspring per mated female; (c) Total number of offspring sired; (d) Relationship between number of mates and the total number of offspring sired by each male. Each transparent dot represents one male. In a–c, solid symbols represent the mean ± SE. In (d), solid line represents model predictions, with their 95% confidence interval indicated by shadow. The slope of relationship for infected males is significantly lower than that for the sham‐treated males on Day 5 post treatment (*p* = .039, adjusted *p* = .116).

No male sired offspring with all 10 virgin females within the mating period, implying that this number of available mates was sufficient to assess the males' maximum reproductive potential. Most males in the experiment successfully inseminated four to eight females; only one male had productive matings with three females and two with nine females. The number of females successfully inseminated by the male (i.e., those that produced at least one offspring, referred to as number of mates) and the number of offspring per mated female (Figure 4a,b) are two key factors contributing to male overall reproductive output. Although both components showed a trend for lower means in infected males (Estimated Marginal Means (EMM) ± SE, proportion of mated females: infected 59.5 ± 1.8%, sham‐treated 63.3 ± 1.8%; number of offspring per mated female, infected 37.4 ± 0.681, sham‐treated 39.1 ± 0.696), neither difference was statistically significant (Table S6). Nonetheless, when the two components were combined in a measure of total offspring production, infected males sired on average 10.6% fewer offspring compared to sham‐treated males (EMM ± SE, infected, 220 ± 5.5, sham‐treated, 246 ± 5.6; treatment, $\chi_{1}^{2}$ = 11.4, *p* < .001; Figure 4c). We did not detect any significant interaction between treatment and DPT (Table S6), indicating that the effects of infection did not change significantly as infection advanced.

Although we did not detect any three‐way interaction between DPT, treatment and number of mates on number of offspring (Table S6), the relationship between the number of mates and the number of offspring (i.e., the Bateman gradient) appeared to differ between infected and sham‐treated males on Day 5 post treatment (Figure 4d; pairwise comparison, *p* = .039, adjusted *p* = .116). Specifically, we observed that infected males had a significantly flatter slope than sham‐treated males, suggesting a decrease in efficiency of male translation of mating opportunities into actual offspring as the number of mating increases.

### 3.5 Replenishment of seminal fluid proteins

**FIGURE 5:**
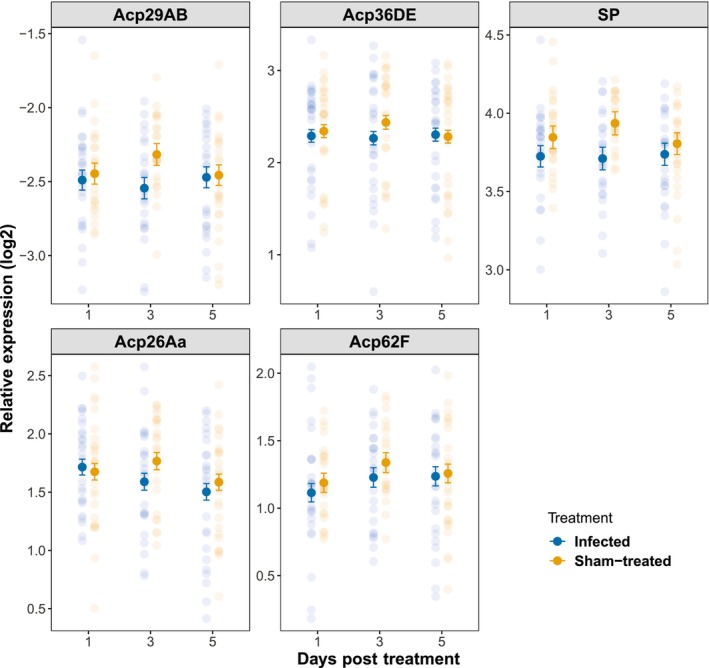
Relative expression of seminal fluid protein genes in infected and sham‐treated males after repeated mating (a proxy for SFP replenishment). Each transparent dot represents one male. Symbols are estimated marginal means ± SE.

Despite their different overall expression levels (*SP* > *Acp36DE* > *Acp26Aa* > *Acp62F* > *Acp29AB*), the five seminal fluid proteins (SFPs) demonstrated consistent gene expression differences between infected and sham‐treated males (treatment × SFP, $\chi_{4}^{2}$ = 2.1, *p* = .71, treatment × SFP × day post treatment, $\chi_{8}^{2}$ = 3.9, *p* = .86; Table S7). In general, infected males had a lower level of SFP expression compared to sham‐treated males (treatment $\chi_{1}^{2}$ = 4.1, *p* = .042; Figure 5). However, the impact of infection on SFP expression did not seem to increase with time post infection as indicated by the insignificant two‐way interaction between day post treatment and treatment (day post treatment × treatment, $\chi_{2}^{2}$ = 2.3, *p* = .32; Figure 5).

**FIGURE 6:**
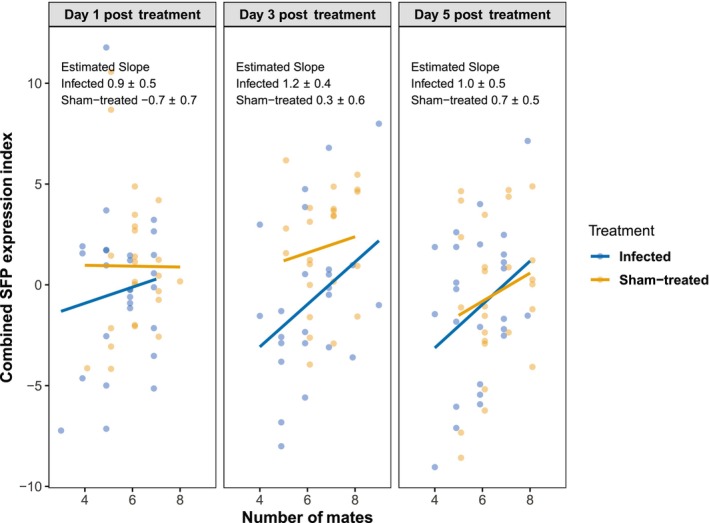
Relationship between number of mates and combined SFP expression index for the infected and sham‐treated males. Each dot represents one male. Solid lines represent predicted values from the linear mixed models; estimated slopes ±SE are indicated.

Males whose SFP expression was measured had mated with multiple females on the previous day, and infected males tended to have a smaller number of mates (see Figure 4). Thus, rather than reduced ability to invest in SFPs, their lower SFP expression might have been driven by a lower SFP depletion due to having less mating events. To address this possibility, we analyzed the relationship between investment in SFPs (quantified as a combined SFP expression index, see Section 2) and the number of mates from the previous day. This relationship had a different slope for infected and sham‐treated males (treatment × number of mates, $\chi_{1}^{2}$ = 4.5, *p* = .034; Figure 6; Table S8). In general, for infected males, the overall SFP expression increased with the number of mates (*t*_76_ = 3.8, *p* < .001; Table S9), while no consistent relationship was detected for sham‐treated males (*t*_73_ = 0.8, *p* = .46; Table S9). Among males that achieved few matings, the infected males had seemingly lower SFP expression index than sham‐treated males, but the difference vanished among males that were more sexually successful. At the point corresponding to the mean mating success (mean number of mates = 6.1), the predicted SFP expression index value was lower for infected than sham‐treated males (treatment, $\chi_{1}^{2}$ = 5.4, *p* = .020; Table S9).

## 4 DISCUSSION

Consistent with reported lethality to a broad range of insects (St. Leger & Wang, 2020), infection by *Metarhizium brunneum* induced high adult morality in our *D. melanogaster* population. Females were more susceptible than males, a finding that aligns with the male‐biased survival observed in previous studies involving fungal infection of *Drosophila* with *Beauveria bassiana* (Shahrestani et al., 2018, 2021; Taylor & Kimbrell, 2007) and *M. anisopliae* (Lu et al., 2015). Mortality did not occur until several days after infection, as has been shown for other *D. melanogaster* populations (Wang et al., 2017) and other insect species (Clifton et al., 2019; Cossentine et al., 2016).

Yet, within 38 hours of infection the host immune system was already strongly activated and continued to mount an increasing response, as indicated by the increasing level of *Drosomycin* expression, reaching more than 100‐fold the level of sham‐treated flies. This increasing level of immune responses over time is consistent with continuing fungal proliferation within the host. While the course of mortality following fungal infection was dose‐dependent, the degree of the immune response—at least in terms of AMP gene expression—appeared not to be. This implies that the spore concentration (10^7^ spores/mL, LT_50_ for males 9 days post infection) used in the remaining experiments was sufficient to induce the maximum level of host immune response against fungal infection. Previous studies looking at the *Diptericin A* expression (active against Gram‐negative bacteria) after either injection or natural infection with fungi have shown that *Diptericin A* is also strongly induced by the fungal challenge (Hedengren‐Olcott et al., 2004; Lemaitre et al., 1997), even if *Diptericin A* does not appear to contribute any antifungal activity (Tzou, Reichhart, et al., 2002). Yet, in our experiment, the increase of *Diptericin A* was only seen at a later stage of the infection and was relatively small (about 2‐fold that of sham controls). This suggests that the immune response to *M. brunneum* in our *D. melanogaster* population was largely confined to the Toll pathway, with little activation of the IMD pathway, as expected in general for fungal infections (Lemaitre & Hoffmann, 2007).

The fungal infection significantly reduced male reproductive potential, quantified as total reproductive output in the absence of rival males and with surplus of potential mates. This may be a result of less available resources after allocating to immune responses and being exploited by the fungus (Cressler et al., 2014). However, the reduction of reproduction success (~11%) reported here was rather small compared with other studies showing the negative relationship between parasitism and male reproductive success, for instance about 56% reduction reported for Taiwan field mice infested by mites (Lin et al., 2014) and about 42% for tapeworm‐infected grain beetles (Worden et al., 2000). Meanwhile, several studies conducted under similar non‐competitive settings did not find a significant reduction in reproductive success following infection (Gao et al., 2021; Rittschof et al., 2013). The two components of the overall reproductive success, number of mates and number of offspring per mated females, both tended to be lower in the infected males, but neither trend was significant, suggesting that they may have contributed to a similar degree to the reduced overall reproductive output. As indicated by the similar mating latency and the comparable number of mates, females did not discriminate strongly against infected males as potential mates, at least in the absence of alternatives. This implies that despite investing in a strong immune response, infected males still managed to provide a satisfying courtship display (Rose et al., 2022) and did not emit any aversive sensory (e.g., olfactory) cues.

The fungus growing within the host not only consumes host resources but also inflicts host damage by releasing metabolites like toxins (Butt et al., 2016; St. Leger & Wang, 2020). Particularly approaching the end of the fungal proliferation, filamentous growth starts and causes serious tissue damage to the host (Castrillo et al., 2005; Hajek & St. Leger, 1994). One would expect that if there were negative impacts of infection on males, the effects would appear several days before death and be more profound at the later stage of the infection. Contrary to this prediction, we found no evidence of increasing negative effects of the infection over time, affecting neither total reproductive output nor its two key components. Nevertheless, we still detected some signs of declining performance of infected males appearing progressively as the infection advanced. The average time taken for infected males to convince female to mate somewhat increased, suggesting a lower sexual performance over the days. Moreover, compared to the sham‐treated males, infected males exhibited a shallower increase in the number of offspring sired as the number of mates increased at the end of the incubation period. Although no mortality was observed during the incubation period, some infected males may be approaching death at the end of this period. This less efficient conversion of mating success to offspring may be a result of faster depletion of sperm or more likely seminal fluid proteins in infected males: SFPs are typically depleted before sperm in *Drosophila* (Hihara, 1981; Hopkins et al., 2019). A previous study has shown that approximately 30%–35% of the SFPs is transferred to female at the first mating (Ravi Ram et al., 2005) and Sirot et al. (2009) have demonstrated a significant decrease in SFP transfer during three successive matings. Traits like the ability to restock SFPs are important in keeping the reproduction machine functioning effectively as SFP depletion will lead to substantially decreased male fertility and paternity assurance (Hihara, 1981; Linklater et al., 2007). Thus, upon repeated mating observed in our experiment (some males mated with up to 9 females), males must replenish his supply of SFPs during and after repeated mating to maintain a high level of fertility.

In general, infected males had lower SFP expression than the sham‐treated males after repeated mating. Although the five SFPs examined in our study vary in function and abundance, they showed a similar pattern of difference, which is consistent with the fact that SFPs have coordinated gene expression (Mohorianu et al., 2018). Although advancement of infection (represented by day post treatment in the analysis) did not affect the relationship between number of mates and overall SFP expression, we found a significant difference in this relationship when comparing infected males and sham‐treated males. For infected males, SFP expression was positively correlated with number of females inseminated by the male on the previous day. While this is consistent with males that mated more having to invest more in SFP replenishment, this relationship was not observed in sham‐treated males. Furthermore, the difference between infected and sham males in SFP expression is most pronounced in males that inseminated the smallest number of females. A more parsimonious explanation is that infected males varied in the degree to which they were affected by the infection. Those that could buffer the physiological cost of infection well could both obtain more mates and induce high SFP expression, similar to non‐infected males, whereas those in poor condition had low mating success and could only afford low SFP expression.

To our knowledge, how infection affects SFP gene expression after repeated mating over the course of infection has never been reported in *Drosophila*. However, change in quantity and quality of SFPs has been reported upon other stressful scenarios. For example, prolonged mite infestation leads to reduced SFP expression, a pattern not evident after brief exposure or in uninfected controls (Benoit et al., 2020). Additionally, it has been shown that as age advances, gene expression of the five representative SFPs decreases and functions (and potentially quality) of SFPs also declines, both of which were accompanied by decreased male reproductive success (Koppik & Fricke, 2017; Sepil et al., 2020). Likewise, the reduced levels of SFP expression in infected males observed in our study may hinder their ability to stimulate female egg production and impair their competitiveness in sperm competition against other males, ultimately leading to lower reproductive success. (Hopkins et al., 2019; Perry et al., 2013; Wigby et al., 2020).

Altogether, the negative effects of fungal infection on male fertility and associated traits in our study were rather mild to undetectable compared with the level of mortality induced by the infection, and they did not markedly increase from Day 1 to Day 5 post infection—even though by Days 7–8 many males would be dead. There are two potential explanations: (1) the infection initially develops slowly and the physiological burden of disease remains low until shortly before death, as shown in Lu et al. (2015) and/or (2) the males compensate by sacrificing other potential future function, as predicted by terminal investment hypothesis. Lu et al. (2015) have shown that fungal load sharply increases in the day preceding death and that flies of *Metarhizium‐*resistant genotype are able to delay the start of the fungal proliferation. While our data do not allow us to distinguish between these explanations, the course of AMP expression indicates that the infection is a burden from early on, if not in terms of damage by the fungus itself, then at least in terms of costs of activation of immune defense, whether due to costs of synthesis of antimicrobial peptides (Gupta et al., 2022) or collateral damage (Bou Sleiman et al., 2015). It has been reported that virgin *D. melanogaster* females strongly upregulate the production of antimicrobial peptides in response to infection with a Gram‐negative bacterium (*Providencia*), while this is not seen in reproductively active females, which leads to their much faster mortality (Gupta et al., 2022). This response, seeming to be pathological in this infection context (Gupta et al., 2022), may represent an overreaction of a system evolved to balance the needs of immune defense and current reproduction, as opposed to the maximum activation of immune system in virgin females. It is tempting to speculate that in the case of *M. brunneum* infection, during the early phases, infected males may also largely compensate for negative effects of the pathogen infection to maintain mating ability and fertility, at the cost of precipitous mortality once a threshold is reached. If so, there would be little additional loss of reproductive fitness during early stages of infection, suggesting that selection for resistance is in this case almost entirely mediated by mortality. However, while this result was unexpected, it still leaves scope for sexual selection to contribute to selection for resistance, particularly if the mild effects we observed become magnified in scenarios where multiple males compete for and are chosen by females.

## AUTHOR CONTRIBUTIONS

**Aijuan Liao:** Conceptualization (equal); data curation (lead); formal analysis (lead); investigation (lead); visualization (lead); writing – original draft (lead); writing – review and editing (equal). **Fanny Cavigliasso:** Data curation (supporting); formal analysis (supporting); investigation (supporting); writing – review and editing (equal). **Loriane Savary:** Data curation (supporting); writing – review and editing (supporting). **Tadeusz J. Kawecki:** Conceptualization (equal); formal analysis (equal); funding acquisition (lead); supervision (lead); writing – review and editing (equal).

## CONFLICT OF INTEREST STATEMENT

The authors have declared no competing interests.

### OPEN RESEARCH BADGES

This article has earned an Open Data badge for making publicly available the digitally‐shareable data necessary to reproduce the reported results. The data is available at https://doi.org/10.5281/zenodo.10132327.

## Supporting information


Figure S1

## Data Availability

Data and R scripts are available on Zenodo (https://doi.org/10.5281/zenodo.10132327).
