# Supplementary material for: Effects of an entomopathogenic fungus on the reproductive potential of Drosophila males
Source: Ecol Evol. 2024 Apr 8;14(4):e11242. doi: 10.1002/ece3.11242 (PMC10999951; doi:10.1002/ece3.11242)
Supplement: Supplementary file 1 — Figure S1 [file ECE3-14-e11242-s001.pdf]

Supplementary Information for article *Effects of an Entomopathogenic Fungus on the Reproductive Potential of Drosophila Males* by Aijuan Liao, Fanny Cavigliasso, Loriane Savary and Tadeusz J. Kawecki

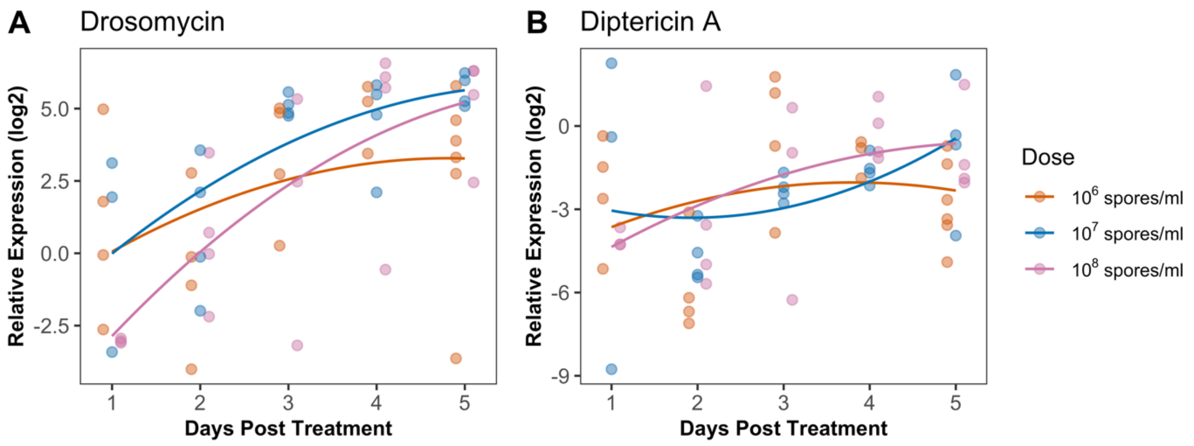

Figure S1 **Immune gene expression is not dose-dependent**. Relative expression (log_2 transformed) of **A:** *Drosomycin* and **B**: *Diptericin A*. Plotted data were from **Immune Assay 2** described in *Activation of Immune System*. Each dot represents a sample with 2-3 males. Solid lines demonstrate the predicted values from the linear mixed models.

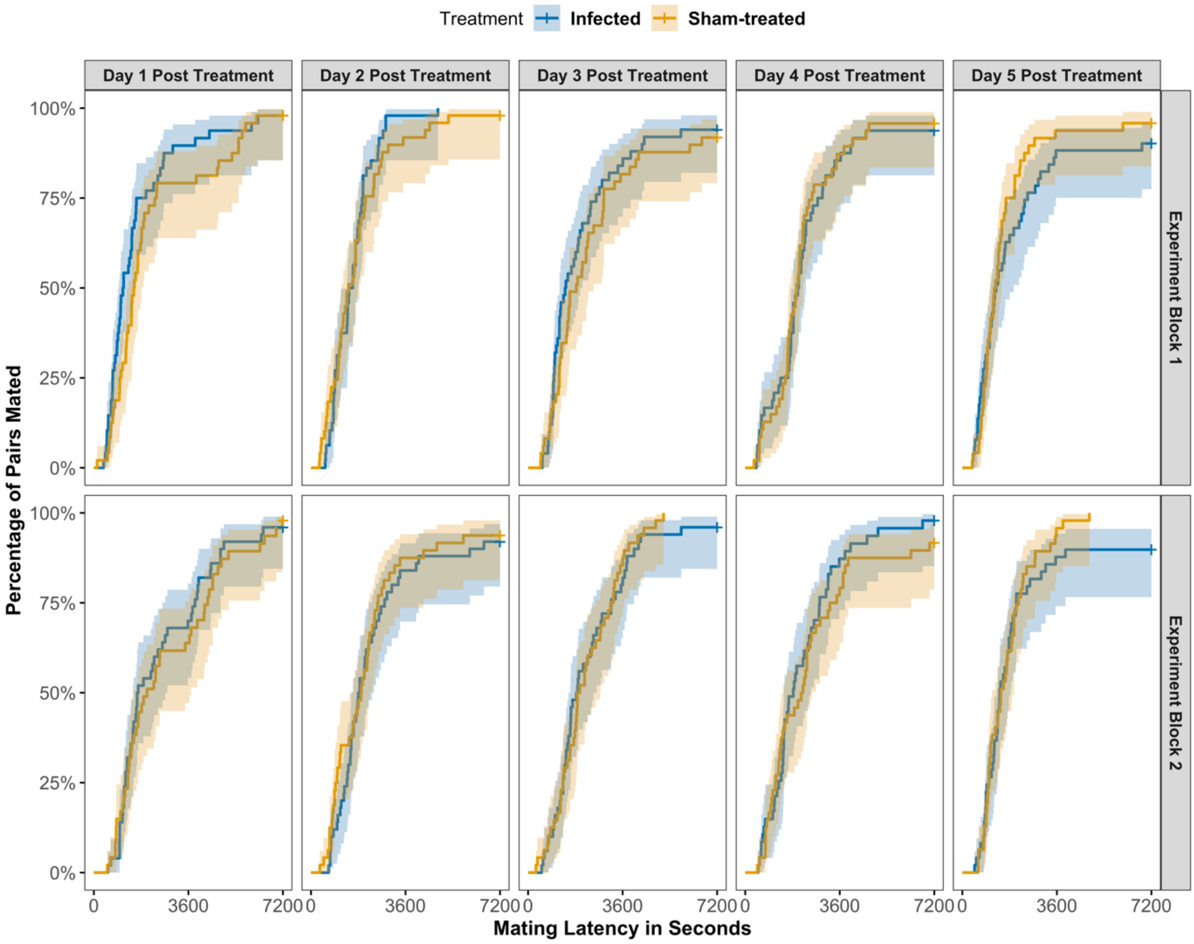

Figure S2 Mating latency of a virgin female (a proxy for male's sexual performance) when paired with an infected or a sham-treated male. Cumulative proportion of pairs that initiated copulation during the 2h observation period. Shadow indicates the 95% confidence interval predicted by the Kaplan-Meier model.

Table S1 Primer overview

	FlyBase ID	Sequence Forward Primer (5' -3')	Sequence Reverse Primer (5' -3')	Reference
**Reference Gene**				
RpL32	FBgn0002626	ATGCTAAGCTGTCGCACAAATG	GTTCGATCCGTAACCGATGT	
eEF1α2	FBgn0000557	GCGTGGGTTTGTGATCAGTT	GATCTTCTCCTTGCCCATCC	
αTub84B	FBgn0003884	TGTCGCGTGTGAAACACTTC	AGCAGGCGTTTCCAATCTG	Ponton et al. 2011
**Immune Gene**				
Drosomycin	FBgn0283461	CGTGAGAACCTTTTCCAATATGAT	TCCCAGGACCACCAGCAT	
Diptericin A	FBgn0004240	GCTGCGCAATCGCTTCTACT	TGGTGGAGTGGGCTTCATG	Leulier et al, 2003
**Seminal Fluid Protein Gene**				
Acp29AB	FBgn0015583	CCACAAACGCCGCAAAATAC	AACGGCTGAAGCTGGATTTC	
SP	FBgn0003034	TTCTTGGTTCTCGTTTGCGT	CTTATCACGAGGATTGGGGC	
Acp26Aa	FBgn0002855	GCTCTCCAATTTTACTGCTGC	TCGCCCTTTTTCGCATCTTT	
Acp36DE	FBgn0011559	TGGTGCCCAGTGAGTCTTTT	TGTGAAGACTCGGGCTTTGG	
Acp62F	FBgn0020509	GACGGAGTGTCCTGTAGCAT	TATCCCGGCTTACACACACA	Koppok and Fricke, 2017

Table S2 Effects of the fixed factors based on the Likelihood Ratio Test. The generalized linear mixed model (binomial distribution, logit link) analyzing the post infection survival includes day post infection, dose, sex and their interactions as fixed factors and vial identity as the random factor. Significant effects ($p \leq 0.05$) are highlighted in bold.

Term	Chisq	Chi Df	Pr(>Chisq)
**Day Post Infection**	**2245.5**	**1**	**< 0.001**
**Dose**	**99.8**	**2**	**< 0.001**
**Sex**	**33.4**	**1**	**< 0.001**
**Day Post Infection × Dose**	**238.3**	**2**	**< 0.001**
**Day Post Infection × Sex**	**8.5**	**1**	**0.004**
Dose × Sex	5.3	2	0.071
**Day Post Infection × Dose × Sex**	**23.6**	**2**	**< 0.001**

Table S3 Effects of the fixed factors on the expression of AMPs based on the Likelihood Ratio Test. The linear mixed model predicting the relative gene expression of *Drosomycin* and *Diptericin A* (Infected males were treated with $\mathbf{10^7}$ spores/ml *M.brunneum*; Data from **Immune Assay 1**). Significant effects ($p \leq 0.05$) are highlighted in bold.

Term	Chisq	Chi Df	Pr(>Chisq)
Diptericin A			
**Treatment**	**4.3**	**1**	**0.039**
**Day Post Treatment**	**39.8**	**1**	**< 0.001**
I(Day Post Treatment^2)	0.8	1	0.383
**Treatment × Day Post Treatment**	**7.8**	**1**	**0.005**
Treatment × I(Day Post Treatment^2)	0.8	1	0.364
**Drosomycin**			
**Treatment**	**80.5**	**1**	**< 0.001**
**Day Post Treatment**	**62.1**	**1**	**< 0.001**
I(Day Post Treatment^2)	0.3	1	0.612
**Treatment × Day Post Treatment**	**59.2**	**1**	**< 0.001**
**Treatment × I(Day Post Treatment^2)**	**7.8**	**1**	**0.005**

Table S4 Effects of the fixed factors based on the Likelihood Ratio Test. The linear mixed model predicting the relative gene expression of *Drosomycin* and *Diptericin A* (Infected males were treated with **10^6, 10^7, 10^8** spores/ml *M.brunneum*; Data from **Immune Assay 2**). Significant effects ($p \leq 0.05$) are highlighted in bold.

Term	Chisq	Chi Df	Pr(>Chisq)
Diptericin A			
Dose	1.2	2	0.550
**Day Post Infection**	**8.6**	**1**	**0.003**
I(Day Post Infection^2)	0.0	1	0.869
Dose × Day Post Infection	1.6	2	0.442
Dose × I(Day Post Infection^2)	1.7	2	0.423
Drosomycin			
Dose	1.7	2	0.429
**Day Post Infection**	**28.0**	**1**	**< 0.001**
I(Day Post Infection^2)	1.8	1	0.181
Dose × Day Post Infection	4.9	2	0.087
Dose × I(Day Post Infection^2)	0.0	2	0.987

Table S5 Effects of the fixed factors of the Cox's proportional hazard mixed effect model. Significant effects ($p \leq 0.05$) are highlighted in bold.

Term	Chi Df	Chisq	Pr(>Chisq)
Treatment	1	0.0	0.867
Day Post Treatment	1	0.5	0.470
Experiment Block	1	3.8	0.052
**Treatment × Day Post Treatment**	**1**	**4.5**	**0.034**

Table S6 Effects of the fixed factors in (generalized) linear mixed models analyzing different components of the male's reproductive success based on the Likelihood Ratio Test. Significant effects ($p \leq 0.05$) are highlighted in bold.

Term	Chisq	Chi Df	Pr(>Chisq)
**Number of Offspring**			
**Day Post Treatment**	**17.3**	**2**	**<0.001**
**Treatment**	**11.4**	**1**	**<0.001**
Experiment Block	0.1	1	0.773
Day Post Treatment × Treatment	0.2	2	0.920
**Number of Mates**			
Day Post Treatment	3.7	2	0.156
Treatment	2.4	1	0.125
Experiment Block	0.0	1	0.968
Day Post Treatment × Treatment	0.1	2	0.973
**Number of Offspring per Mated Female**			
**Day Post Treatment**	**20.2**	**2**	**<0.001**
Treatment	3.1	1	0.077
Experiment Block	0.2	1	0.639
Day Post Treatment × Treatment	0.2	2	0.921
**Number of Offspring (model including Number of Mates)**			
**DPT**	**21.6**	**2**	**<0.001**
**Treatment**	**6.4**	**1**	**0.011**
**Number of Mates**	**95.1**	**1**	**<0.001**
Experiment Block	0.5	1	0.499
Day Post Treatment × Treatment	0.5	2	0.792
Day Post Treatment × Number of Mates	3.5	2	0.171
Treatment × Number of Mates	0.4	1	0.516
Day Post Treatment × Treatment × Number of Mates	4.7	2	0.098

Table S7 Effects of the fixed factors based on the Likelihood Ratio Test. The linear mixed model predicting the relative gene expression of seminal fluid protein (SFP) is with the following independent variables: day post treatment (DPT), identity of SFPs, male treatment (infected vs. sham-treated) and all the possible two-way and three-way interactions of three variables and experiment block (n=2) as the fixed factors and male identity as the random factor. Significant effects ($p \leq 0.05$) are highlighted in bold.

Term	Chisq	Chi Df	Pr(>Chisq)
DPT	1.4	2	0.491
**Treatment**	**4.1**	**1**	**0.042**
**SFP**	**2924.4**	**4**	**<0.001**
**Experiment Block**	**83.0**	**1**	**<0.001**
DPT × Treatment	2.3	2	0.317
DPT × SFP	13.4	8	0.099
Treatment × SFP	2.1	4	0.711
DPT × Treatment × SFP	3.9	8	0.863

Table S8 Effects of the fixed factors on the combined SFP expression index based on the Likelihood Ratio Test. The linear mixed model predicting values of the combined SFP expression index is with the following independent variables: day post treatment (DPT), number of mates, treatment (infected vs. sham-treated), their interactions and experiment block as fixed factors, and day of experiment as the random factor. Significant effects ($p \leq 0.05$) are highlighted in bold.

Term	Chisq	Chi Df	Pr(>Chisq)
**Model with Number of Mates**			
DPT	1.72	2	0.422
**Treatment**	**5.4**	**1**	**0.020**
**Number of Mates**	**6.4**	**1**	**0.011**
**Experiment Block**	**21.7**	**1**	**<0.001**
DPT×Treatment	3.8	2	0.147
DPT×Number of Mates	1.9	2	0.378
**Treatment×Number of Mates**	**4.5**	**1**	**0.034**
DPT×Treatment×Number of Mates	1.4	2	0.488

Table S9 Estimated slopes of the relationship between number of mates and the combined SFP expression index for infected and sham-treated males. Data are from males of all three days post treatment. Linear mixed models used for extracting the estimated coefficients are with combined SFP expression index as response variable, the number of mates and experiment block as the fixed factors and day of experiment as the random factor. Intercepts represent the estimates when the number of mates is at the mean level (mean number of mates=6.1). Significant terms (except intercept) are highlighted in bold.

Estimates of Coefficients

Male Treatment		Estimate	Std. Error	df	t value	Pr(>|t|)
Infected	(Intercept)	1.97	0.47	76	4.15	<0.001
	**Block3**	**-4.74**	**0.65**	**76**	**-7.26**	**<0.001**
	**Number of Mates**	**0.99**	**0.26**	**76**	**3.75**	**<0.001**
Sham-treated	(Intercept)	2.69	0.53	8.01	5.07	<0.001
	**Block3**	**-3.92**	**0.73**	**6.96**	**-5.40**	**0.001**
	Number of Mates	0.25	0.34	72.68	0.75	0.458
